# Role of microorganisms isolated from cows with mastitis in Moscow region in biofilm formation

**DOI:** 10.14202/vetworld.2021.40-48

**Published:** 2021-01-07

**Authors:** Pavel Rudenko, Nadezhda Sachivkina, Yury Vatnikov, Sergey Shabunin, Sergey Engashev, Svetlana Kontsevaya, Arfenia Karamyan, Dmitry Bokov, Olga Kuznetsova, Elena Vasilieva

**Affiliations:** 1 Biological Testing Laboratory, Branch of Shemyakin-Ovchinnikov Institute of Bioorganic Chemistry of the Russian Academy of Sciences, Moscow Region, Russia; 2 Department of Veterinary Medicine, Peoples’ Friendship University of Russia, Moscow, Russia; 3 Department of Microbiology and Virology, Medical Institute, Peoples’ Friendship University of Russia, Moscow, Russia; 4 Russian Research Veterinary Institute of Pathology, Pharmacology, and Therapy of the Russian Academy of Agricultural Sciences, Voronezh, Russia; 5 Department of Parasitology and Veterinary and Sanitary Expertise, Moscow State Academy of Veterinary Medicine and Biotechnology – MVA named after K.I. Skryabin, Moscow, Russia; 6 Department of Non-communicable Diseases, Belgorod State Agricultural University, Belgorod, Russia; 7 Institute of Pharmacy, Sechenov First Moscow State Medical University, Moscow, Russia; 8 Department of Biochemistry, Medical Institute, Peoples’ Friendship University of Russia, Moscow, Russia

**Keywords:** bacterial communities, biofilms, biogeocenosis, cows, mastitis, sensitivity to antibiotics

## Abstract

**Background and Aim:**

Mastitis is one of the most important diseases of cows and the most expensive pathology for the dairy industry. Therefore, this study was conducted to explore the role of microorganisms isolated from cows with mastitis in the formation of biofilms under the conditions of farm biogeocenosis in the Moscow region.

**Materials and Methods:**

Periodic visits to 12 farms in the Moscow region were conducted to explore the microbial profile of the udder of cows with mastitis. During the visits, 103 milk samples from sick animals were collected and examined. Through microbiological analyses, 486 cultures of microorganisms were identified, which are assigned to 11 genera. Mastitis in cows is caused not only by a single pathogen but also by microbial associations, which included two to seven microbial isolates.

**Results:**

It was observed that 309 isolates (63.6%) from the total number of isolated microorganisms could form a biofilm. The ability to form biofilms was most frequently observed in *Staphylococcus aureus* (18.8%), *Escherichia coli* (11.9%), and *Staphylococcus uberis* (11.7%) cultures from the total number of biofilm-forming microbial cultures. Low biofilm-forming ability among the isolated microorganisms was found in lactobacilli, wherein only 20 (22.5%) *Lactobacillus* strains had the ability to form biofilms. The isolated microorganisms exhibited different sensitivities to antimicrobial agents, which cause difficulty in selecting an antimicrobial agent that would act on all aspects of the parasitocenosis.

**Conclusion:**

A high proportion of microorganisms isolated from cows with mastitis have the ability to form biofilms. The isolated microorganisms exhibited different and highly heterogeneous sensitivity to the action of antimicrobial drugs. This causes difficulty in using these tools for the effective control of mastitis in cows, which is frequently caused by pathogenic associations of microbial biofilms. Therefore, it is important to explore novel and more effective methods to combat this disease.

## Introduction

Veterinary medicine plays a significant role in the quality of life of people. One of the most important national tasks is to improve veterinary services and reduces the morbidity and death of animals. In recent years, veterinary medicine has achieved adequate success in its development [1-3]. However, despite the division of cattle farms in the process of agricultural reform, obstetric, and gynecological diseases in farm animals remain the leading problem for veterinary medicine specialists. This is due to the fact that the traditional milk production technology is used in reorganized collective farms and biogeocenosis has been preserved in the process of evolution, including the associations of conditionally pathogenic bacteria that cause different pathological processes in animals under farm conditions [4-8].

In artificial biogeocenosis, conditionally pathogenic microflora that circulates in the farm can cause various associated diseases in farm animals. These diseases include mastitis, endometritis, vaginitis in adult animals, and gastrointestinal and respiratory diseases in newborn young animals [9-13].

Mastitis in cows is one of the most important diseases of cattle and the most expensive pathology for the milk industry. In cattle, it causes significant economic losses in the dairy industry, which is a serious problem throughout the world. To date, the etiology of mastitis remained controversial. Therefore, most of the authors believe that the cause of udder inflammation is microflora. However, some researchers emphasize the action of mechanical and chemical factors that initially cause breast injury, only after which the microbial factor begins to act [14-18]. Furthermore, animals with subclinical mastitis are an important and frequently neglected reservoir for the transmission of the association of microorganisms within the cattle farm or between animal herds. However, they may carry virulence and antibiotic resistance genes that contribute to persistent colonization, hinder with mastitis control in farm biogeocenosis, and may create public health risks [19].

Researchers have identified approximately 100 different types of microorganisms from the milk of cows with mastitis, such as bacteria, viruses, and microscopic fungi, which are attributed to the etiology of this disease. The following microorganisms have been isolated from cows with mastitis: Enterobacteria (*Escherichia coli*, *Klebsiella* *pneumoniae*, *Klebsiella oxytoca*, and *Salmonella*), *Pseudomonas*, Streptococci (primarily *Streptococcus agalactiae*, *Streptococcus dysgalactiae*, *Staphylococcus aureus* (18.8%), *E. coli* (11.9%), and *Staphylococcus uberis*), Staphylococci (primarily *S. aureus*), as well as *Enterococcus*, *Corynebacterium*, *Pasteurella*, *Nocardia*, *Sarcina*, *Mycoplasma*, *Brucella*, *Clostridium*, *Listeria*, some viruses, and fungi from the genus *Candida* [10,20-22]. In general, mastitis is caused not only by one microorganism but also by an association of microorganisms, and the primary causative agents of mastitis are Staphylococci and Streptococci [23].

Recently, there has been extensive interest in reports about the ability of microorganisms to form biofilms. The vast majority of bacteria that live in natural ecosystems exist in an immobilized state, in which microbial cells are fixed onto a solid surface and tightly pressed together, forming specific formations termed biofilms. Subsequently, it was established that along with the resident microflora of different biotopes of the body in the form of biofilms, there are pathogenic microorganisms. Biofilm is a living, constantly updating community of microorganisms that are irreversibly attached to a biogenic or abiogenic substrate and to each other, surrounded by an extracellular polymer matrix, which is produced by the same bacteria and protects them against ­aggressive environmental effects. Such bacterial communities can be formed by one or more species of bacteria and consist of both actively functioning cells and dormant or uncultivated forms [24].

The formation of biofilms in cows with mastitis is considered as a selective advantage for pathogenic microorganisms, which contributes to the ­conservation of bacteria in the udder. The biofilm is an important virulence factor in mastitis, and as a result, the focused infectious origin becomes more challenging to treat and eradicate, which makes this problem more relevant. Biofilms in the udders of cows with mastitis reduce the effect of antibiotics and enable microorganisms to evade the innate immune system. This can result in permanent or relapsing infections in the herd [25-27].

In any associated disease, various complications occur more frequently, and their diagnosis, as well as the choice of treatment and the methods to combat them, becomes difficult. The analysis of literature sources allows us to identify the following areas for controlling animal diseases that are caused by conditionally pathogenic microflora: Use of antibiotics and other antibacterial drugs, use of probiotics, use of vaccines, and breaking the epizootic chain by replacing maternity wards and dispensaries, a comprehensive method.

One of the traditional methods of treating cows with mastitis is the use of antibiotics. Unsystematic and empirical use of antibiotics for treating mastitis in cows often leads to violations of the microbial profile and the occurrence of new antibiotic-resistant strains of pathogenic bacteria. Moreover, antibiotic therapy for mastitis promotes the accumulation of antimicrobial drugs in milk, which has a negative impact on its quality. Antibiotics also kill most of the bacteria that are present in the digestive tract and represent the body’s colonization resistance, which causes an imbalance in the intestinal microbiome and destroys the ecosystem that is commonly present in the intestine of a healthy animal [28-31]. Furthermore, due to the wide range of pathogens in cows with mastitis, the use of commercial vaccines has demonstrated low effectiveness [32-38]. This situation demands a more detailed study on the etiology and pathogenesis of mastitis in cows in farm biogeocenosis, as well as an immediate search for alternative and evolutionarily reasonable methods of treatment.

Therefore, this study was conducted to explore the role of microorganisms isolated from cows with mastitis in the formation of biofilms under the conditions of farm biogeocenosis in the Moscow region.

## Materials and Methods

### Ethical approval

All samples were taken from cows in accordance with the regulations for the care and husbandry concerning experimental animals and approved by the Animal Care Committee of Animal Health Research Institute of Bioorganic Chemistry of the Russian Academy of Sciences, Russia.

### Samples

The bacterial etiology of mastitis in cows was investigated in 12 farms in the Moscow region from July 2019 to June 2020 with a population of 12,254 cattle, including 4,445 cows. For this purpose, periodic visits were made to the farms for epizootiological examinations of farm biogeocenosis and for selection of blood serums stabilized with heparin and samples of pathological material to be used for bacteriological, mycological, virological, hematological, and immunological studies.

During the epizootiological examination of farms, the incidence of mastitis in cows was examined. The presence of other infectious diseases (tuberculosis, leukemia, and acute respiratory diseases) in the farms was also investigated. In the bacteriological examination, the set of microorganisms, the presence of the ability to form biofilms, and also their relationships with each other in microbial associations were investigated. By conducting a survey of biogeocenosis of cattle farms, the conditions of keeping, feeding, and exploitation of animals were analyzed in detail.

A clinical study was conducted to diagnose mastitis in cows by collecting details about anamnesis and determination of temperature, pulse, and respiration. First, a general study of the systems was performed, followed by a special study (udder inspection, palpation, trial milking, and organoleptic evaluation of the milked milk).

During the collection of anamnestic data, the state of females was determined (the presence of pregnancy, dry period or the period of childbirth, the course of postpartum period, and the stage of the sexual cycle were diagnosed), including the volume of yield, method of milking, and drugs that were used to treat when the animal was ill, and the personnel and method of providing assistance.

During the examination of the mammary gland, its shape, development, size, and how the nipples are located was examined. Special attention was paid to the size and shape of the symmetrical udder lobes and the state of the skin. Surface palpation was used to determine the local temperature in symmetrical areas of breast lobes. When deep soreness was observed, the presence of foci of softening or compaction, the condition of supraparietal lymph nodes, their size, consistency, and soreness were evaluated. Nipples were examined by rolling with two fingers to detect morphological changes in their wall, and the patency of the channel was studied. Supraparietal lymph nodes were palpated to determine their size, pain, sensitivity, mobility, and consistency.

Bacteriological, mycological, and hematological examinations and determination of some properties of the isolated bacterial cultures were conducted in the bacteriological laboratory for investigating the infection factors in the veterinary Department RUDN. Exclusion of viral agents (infectious rhinotracheitis, parainfluenza, and viral diarrhea) was conducted retrospectively.

Milk samples were collected from cows with mastitis and placed in sterile test tubes. Before sampling the milk, the udder teats were wiped with a swab dipped in 70% ethanol. The first portion of milk (5-10 cm^3^), which was located in the nipple channel, was placed in a separate dish. The subsequent portions of milk were selected for the study.

In total, 103 milk samples were selected from farms in the Moscow region from cows with mastitis for bacteriological examination.

### Bacterial isolation and identification

In the microbiological examination of selected pathological material made crops on nutrient media by Pasteur pipette. For yeast-like fungi, Sabouraud medium was used; for Staphylococci, peptone salt medium, yolk-salt agar, and meat peptone agar were used; for Enterobacteria, Endo medium, Ploskirev medium, and bismuth sulfite agar were used; for Bifidobacteria and Blaurock medium was used; and for lactobacilli, MRS medium, PSL, and skim milk were used. The plates were incubated again in a thermostat at 37-38°C for 24 h, and in the absence of growth, the plates were kept for up to 3 days.

After exploring the cultural and morphological characteristics of all individual colonies, they were transferred to test tubes and incubated at 37-38°C for 24 h. The resulting pure bacterial cultures were tested for mobility in squashed droplet preparations by phase-contrast microscopy in a darkened field of view and subjected to the identification.

The number of microorganisms in 1.0 cm^3^ of milk sample (C) was calculated using the following formula and expressed in logarithms with a base of 10:

* C = (N/V)* × *K*

Where N is the average number of colonies in one bacteriological cup; V is the volume of suspension, which is applied during seeding on the surface of agar; and K is the multiplicity of dilution.

The morphology of bacteria was examined in Gram- and Romanowsky-Giemsa-stained smears. Further identification based on biochemical properties was conducted, according to the “Bergey bacteria Determinant.”

Determination of *E. coli* serogroups was performed using a set of “serum “O”-coli agglutinating.” Determination of serovariants isolated from the pathological material of *Salmonella* cultures was conducted by an agglutination reaction on glass using a set of *Salmonella* O-complex and monoreceptor O-and N-agglutinating serums.

To examine hemolytic activity, pure cultures were isolated and replanted on meat peptone agar with the addition of 5% fresh defibrinated blood of cattle. After 24 h of incubation in a thermostat at 37°C, the presence and nature of hemolysis were recorded. The strains of microorganisms that formed well-marked zones of hemolysis on blood meat peptone agar were considered to be hemolytic.

To determine the pathogenicity of isolated cultures, three white mice weighing 14-16 g were inoculated intraperitoneally with 1 billion microbial cells of each strain of the microorganism. The laboratory animals were observed for 5 days. Cultures were considered to be pathogenic if one or more mice died within 5 days after infection.

In this study, 486 microorganisms that were isolated from milk samples, collected from cows with mastitis, were examined for their ability to form biofilms. The optical density (OD) of a biofilm was measured by the degree of binding of crystal violet (HiMedia, India) at a wavelength of 580 nm (OD580) in an Immunochem-2100 microplate photometric analyzer (HTI, USA). The test samples (control and experiment) were added to the wells of a 96-well plate (Medpolymer, Russia) and cultured at 37°C for 48 h. Then, the liquid was removed, and the wells of plate were washed 3 times with 200 μL of phosphate saline solution (pH 7.3). In each washing step, the plates were shaken for 5 min. The biofilms were placed in 150 μL of a fixative of 96.0% ethanol for 15 min, and then the wells were dried at 37°C for 20 min. The cultivation was performed at 37°C for 5 min by adding a 0.5% solution of crystal violet to the wells. The contents of the wells were removed, rinsed 3 times with 200 μL of phosphate saline solution (pH 7.3), and then dried. Dye was eluted from adherent cells with 200 μL of 96.0% ethanol for 30 min [1]. The examined cultures of microorganisms were considered to be capable of forming biofilms when the OD of the sample was more than 2 times the OD of the control.

The isolated bacterial cultures were analyzed for sensitivity to antibacterial drugs and fungi, wherein sensitivity to antimycotics was determined with using paper disks by a generally accepted method. A total of 14 antibacterial drugs (benzylpenicillin, methicillin, amoxicillin, Cobactan, cephalexin, gentamicin, kanamycin, streptomycin, tetracycline, doxycycline, lincomycin, enrofloxacin norfloxacin, and ofloxacin) and three antimycotic drugs (amphotericin B, fluconazole, and itraconazole) were used. In this study, microorganisms that displayed >18 mm of growth delay around the paper disk were considered as sensitive to the antimicrobial drug, those with 11–18 mm of growth delay were considered to be less sensitive, and strains that showed <10 mm of growth delay were considered as non-sensitive.

### Statistical analysis

The experimental data were processed using descriptive and inferential statistics. Mean and standard deviation of the OD values and adhesive properties were calculated using Microsoft Excel. Differences between mean values of samples and those of control were determined using Student’s t-test, and the statistical significance of differences was set at p≤0.05.

## Results and Discussion

Colostrum and milk promote the establishment and formation of microbial cenosis in the gastrointestinal tract of newborn animals, which are also a factor responsible for the transmission of conditionally pathogenic bacteria from cows to calves during udder inflammation. Hence, mastitis plays a key role in the occurrence and spread of infection factors in farm biogeocenoses, particularly in the distribution of gastrointestinal and respiratory diseases of newborn calves.

To examine the microbial profile of the udder of cows with mastitis, periodic visits were made to 12 farms in the Moscow region. Simultaneously, 103 samples of milk from sick animals were selected and examined. Table-1 shows the results of bacteriological examination of the milk of cows with mastitis.

**Table-1:** Results of microbiological research of cow’s milk, patients with mastitis (n=103).

Type of microorganism	Number of isolates	

	Absolute number	%
*Streptococcus* spp.	125	25.7
*Staphylococcus* spp.	101	20.8
*Salmonella* spp.	5	1.0
*Escherichia* spp.	46	9.6
*Pseudomonas* spp.	23	4.7
*Pasteurella* spp.	8	1.6
*Klebsiella* spp.	10	2.1
*Proteus* spp.	45	9.2
*Bacillus* spp.	23	4.7
*Lactobacillus* spp.	89	18.3
*Candida* spp.	11	2.3
Total	486	100.0

In the microbiological examination, 486 cultures of microorganisms were identified that were assigned to 11 genera. The results also showed that representatives of *Streptococcus* spp., *Staphylococcus* spp., *Lactobacillus* spp., and *Escherichia* spp. were most often isolated, with the identification rates being 25.7%, 20.8%, 18.3%, and 9.6%, respectively, from the udder secretions of cows with mastitis.

Table-2 shows further details of the results of microbiological examination of the species composition of milk of cows with mastitis. As shown in Table-2, the most common pathogens in cows with mastitis are *S. aureus* (n=63, 13.0%), *E. coli* (n=46, 9.5%), and *S. uberis* (n=42, 8.7%). *S. dysgalactiae* and *Proteus vulgaris* (each n=27, 5.6%) and *Pseudomonas aeruginosa* and *Bacillus subtilis* (each n=23, 4.7%) were significantly less isolated among the total number of isolates. One of the pathogens that are involved in the etiology of mastitis in cows in the farm biocenoses of Moscow region was a representative fungus of the genus *Candida*, *Candida albicans*, which was isolated from 11 (2.3%) samples of the udder secretions of cows with mastitis.

**Table-2:** Species analysis of the microbiota of milk cows with mastitis (n=103).

Type of microorganism	Number of isolates	

	Absolute number	%
*Streptococcus agalactiae*	19	3.9
*Streptococcus dysgalactiae*	27	5.6
*Staphylococcus uberis*	42	8.7
*Streptococcus pyogenes*	11	2.3
*Streptococcus pneumoniae*	9	1.8
*Streptococcus faecalis*	17	3.5
*Staphylococcus aureus*	63	13.0
*Staphylococcus saprophyticus*	12	2.5
*Staphylococcus epidermidis*	19	3.9
*Staphylococcus intermedius*	7	1.4
*Salmonella enterica*	5	1.0
*Escherichia coli*	46	9.5
*Pseudomonas aeruginosa*	23	4.7
*Pasteurella multocida*	8	1.6
*Klebsiella pneumoniae*	6	1.2
*Klebsiella ?xytoca*	4	0.8
*Proteus vulgaris*	27	5.6
*Proteus mirabilis*	18	3.7
*Bacillus subtilis*	23	4.7
*Lactiplantibacillus plantarum*	16	3.3
*Lactobacillus rhamnosus*	17	3.5
*Lactobacillus acidophilus*	22	4.5
*Lactobacillus xylosus*	6	1.2
*Lactococcus lactis*	28	5.8
*Candida albicans*	11	2.3
Total	486	100.0

The representatives of lactobacilli isolated from the milk samples of cows with mastitis were categorized as *Lactococcus lactis*, *Lactobacillus acidophilus*, *Lactobacillus rhamnosus*, *Lactiplantibacillus plantarum*, and *Lactobacillus xylosus*, with rates being 5.8%, 4.5%, 3.5%, 3.3%, and 1.2%, respectively, among the total number of isolated cultures of microorganisms.

Table-3 shows the serological typing of the isolated cultures of *E. coli*.

**Table-3:** Serological identification of *Escherichia coli* cultures, allocated from cows with mastitis.

Serogroup	Number of isolates	

	Absolute number	%
О1	2	4.2
О2	1	2.2
О4	3	6.5
О8	7	15.2
О9	1	2.2
О18	7	15.2
О22	1	2.2
О26	4	8.7
О33	1	2.2
О78	6	13.1
О101	5	10.9
О111	3	6.5
О126	5	10.9
Total	46	100.0

In the milk of cows with mastitis, O8 and O18 were the most often isolated (each n=7, 15.2%), followed by O78 (n=6, 13.1%) and O101 and O126 (each n=5, 10.9%), among the total number of isolated *E. coli* serotypes. Moreover, *E. coli* cultures that had hemolytic activity were isolated from cows with mastitis. Hence, 37 (80.4%) isolates among the total number of *E. coli* in the udder secretions of cows with mastitis had hemolysin-producing properties.

Serological identification of three isolated *Salmonella* cultures was also conducted in this study. Two cultures (66.7%) were classified as serovars of *Salmonella* Enteritidis and one culture (33.3%) was classified as a serovar of *Salmonella* Typhimurium.

Table-4 shows the number of microorganisms (lg) in 1 cm^3^ of milk of cows with mastitis. It was observed that the milk of cows with mastitis contained the representatives of the genera *Staphylococcus* spp., *Escherichia* spp., *Pseudomonas* spp., and *Streptococcus* spp. in the highest concentration, at 5.67±0.08, 4.37±0.32, 4.24±0.20, and 4.13±0.15 lg, respectively. *Candida* was observed at varying levels of 10^2^ CFU. It must be noted that representatives of the genus *Lactobacillus* were present only at the concentration of 1.63±0.16 lg, whereas those of the genus *Bifidobacterium* were completely absent.

**Table-4:** Number of microorganisms (lg) in 1 cm^3^ of cows’ udder secretions with mastitis.

Type of microorganism	Number of isolates
*Streptococcus* spp.	4.13±0.15
*Staphylococcus* spp.	5.67±0.08
*Salmonella* spp.	2.94±0.16
*Escherichia* spp.	4.37±0.32
*Pseudomonas* spp.	4.24±0.20
*Pasteurella* spp.	2.51±0.42
*Klebsiella* spp.	1.47±0.15
*Proteus* spp.	3.50±0.09
*Bacillus* spp.	2.97±0.13
*Lactobacillus* spp.	1.63±0.16
*Candida* spp.	2.13±0.36

Table-5 shows the pathogenic properties of all the 486 isolates in the udder secretions of cows with mastitis. The most common pathogenic properties were exhibited by cultures of *S. aureus* (n=48, 27.7%), *E. coli* (n = 22, 12.7%), *S. uberis* (n = 18, 10.3%), and *S. dysgalactiae* (n=17, 9.8%) among the total number of pathogenic isolates in white mice. Of the 11 isolated cultures of fungi of the genus *Candida*, five isolates (2.9%) exhibited pathogenicity. An important finding was that in the surviving white mice, it was not possible to isolate the initial cultures of microorganisms, whereas the pathogen was frequently isolated from dead animals.

**Table-5:** Pathogenicity for white mice that isolated cultures of microorganisms in mastitis.

Type of microorganism	Number of crops from which:			

	Pathogenic		Non-pathogenic	
	
	Absolute number	%	Absolute number	%
*Streptococcus agalactiae* (n=19)	11	6.3	8	2.6
*Streptococcus dysgalactiae* (n=27)	17	9.8	10	3.2
*Staphylococcus uberis* (n=42)	18	10.3	24	7.7
*Streptococcus pyogenes* (n=11)	6	3.4	5	1.6
*S. pneumonia* (n=9)	4	2.3	5	1.6
*Streptococcus faecalis* (n=17)	-	-	17	5.5
*Staphylococcus aureus* (n=63)	48	27.7	15	4.8
*Staphylococcus saprophyticus* (n=12)	-	-	12	3.8
*Staphylococcus epidermidis* (n=19)	-	-	19	6.1
*Staphylococcus intermedius* (n=7)	2	1.1	5	1.6
*Salmonella enterica* (n=5)	5	2.9	-	-
*Escherichia coli* (n=46)	22	12.7	24	7.7
*Pseudomonas aeruginosa* (n=23)	11	6.3	12	3.8
*Pasteurella multocida* (n=8)	5	2.9	3	0.9
*Klebsiella pneumoniae* (n=6)	6	3.4	-	-
*Klebsiella oxytoca* (n=4)	4	2.3	-	-
*Proteus vulgaris* (n=27)	7	4.0	20	6.4
*Proteus mirabilis* (n=18)	3	1.7	15	4.8
*Bacillus subtilis* (n=23)	-	-	23	7.4
*Lactiplantibacillus plantarum* (n=16)	-	-	16	5.1
*Lactobacillus rhamnosus* (n=17)	-	-	17	5.5
*Lactobacillus acidophilus* (n=22)	-	-	22	7.1
*Lactobacillus xylosus* (n=6)	-	-	6	1.9
*Lactococcus lactis* (n=28)	-	-	28	9.0
*Candida albicans* (n=11)	5	2.9	6	1.9
Total (n=486)	174	100.0	312	100.0

Moreover, the isolated cultures of lactobacilli did not possess pathogenic properties. These results confirmed that the formation of biofilm by the communities of microorganisms is one of the primary strategies for the survival of bacteria among the organisms of infected hosts. Therefore, the ability of a microbial agent to form a biofilm is one of the primary factors for pathogenicity. Table-6 shows the results pertaining to the determination of the biofilm formation ability of the isolated microorganisms. A total of 309 (63.6%) isolates among the total number of isolated microorganisms were found to possess biofilm-forming ability. The ability to form biofilms was most frequently observed in the cultures of *S. aureus*, *E. coli*, and *S. uberis*, the rates being 18.8%, 11.9%, and 11.7%, respectively, among the total number of biofilm-forming cultures of microorganisms.

**Table-6:** Assessment of the ability of milk isolates from cows with mastitis to biofilm formation.

Type of microorganism	Indication of biofilm formation	

	Absolute number	%
*Streptococcus agalactiae* (n=19)	11	3.6
*Streptococcus dysgalactiae* (n=27)	19	6.1
*Staphylococcus uberis* (n=42)	36	11.7
*Streptococcus pyogenes* (n=11)	8	2.6
*S. pneumonia* (n=9)	7	2.3
*Streptococcus faecalis* (n=17)	4	1.3
*Staphylococcus aureus* (n=63)	58	18.8
*Staphylococcus saprophyticus* (n=12)	4	1.3
*Staphylococcus epidermidis* (n=19)	3	0.9
*Staphylococcus intermedius* (n=7)	5	1.6
*Salmonella enterica* (n=5)	5	1.6
*Escherichia coli* (n=46)	37	11.9
*Pseudomonas aeruginosa* (n=23)	17	5.5
*Pasteurella multocida* (n=8)	6	1.9
*Klebsiella pneumoniae* (n=6)	4	1.3
*Klebsiella oxytoca* (n=4)	2	0.6
*Proteus vulgaris* (n=27)	27	8.7
*Proteus mirabilis* (n=18)	18	5.8
*Bacillus subtilis* (n=23)	11	3.6
*Lactiplantibacillus plantarum* (n=16)	3	0.9
*Lactobacillus rhamnosus* (n=17)	5	1.6
*Lactobacillus acidophilus* (n=22)	6	1.9
*Lactobacillus xylosus* (n=6)	1	0.3
*Lactococcus lactis* (n=28)	5	1.6
*Candida albicans* (n=11)	7	2.3
Total (n=486)	309	100.0

A low ability of biofilm formation was detected in the isolated representatives of *Lactobacilli*. Only 20 *Lactobacillus* strains (22.5%) among the total number of isolated representatives of the genus *Lactobacillus* had the ability to form biofilms.

Identifying a stable tendency in the global distribution of poly-resistant strains of microorganisms comprises an extremely important factor in mastitis antibiotic therapy, because only the timely administration of an appropriate antibiotic therapy can provide a high treatment effect. Unsystematic and empirical use of antimicrobial agents in farm biocenoses, which is often done in veterinary practice to combat several infection factors in productive animals, results in the occurrence of antibiotic-resistant strains of conditionally pathogenic microorganisms and in the growth of their virulence and pathogenicity.

Table-7 shows the sensitivity results of selected microflora to antimicrobial substances in cows with mastitis. In total, 475 bacteria and 11 fungi were isolated from the udder secretions of cows with mastitis. In this study, 14 antibacterial drugs, benzylpenicillin, methicillin, amoxicillin, Cobactan, cephalexin, gentamicin, kanamycin, streptomycin, tetracycline, doxycycline, lincomycin, enrofloxacin, norfloxacin, and ofloxacin, as well as three antimycotic drugs, amphotericin B, fluconazole, and itraconazole, were used.

**Table-7:** Results of determining the sensitivity of isolated microorganisms in mastitis in cows to antibiotics and antimycotics.

Antibacterial drugs	Indicators of antibiotic sensitivity of isolated microorganisms					

	Sensitive		Low-sensitive		Insensitive	
		
	Absolute number	% of total number of isolates	Absolute number	% of total number of isolates	Absolute Number	% of total number of isolates
Benzylpenicillin	298	62.8	41	8.6	136	28.6
Methicillin	211	44.4	49	10.3	215	45.3
Amoxicillin	373	78.5	24	5.1	78	16.4
Cobactan	438	92.2	12	2.5	25	5.3
Cefalexinum	455	95.8	8	1.7	12	2.5
Gentamicin	306	64.5	91	19.1	78	16.4
Kanamycin	276	58.1	107	22.5	92	19.4
Streptomycin	309	65.1	43	9.1	123	25.8
Tetracycline	306	64.4	67	14.1	102	21.5
Doxycycline	307	64.7	79	16.6	89	18.7
Lincomycin	300	63.1	82	17.3	93	19.6
Enrofloxacin	466	98.1	6	1.3	3	0.6
Norfloxacin	440	92.6	8	1.7	27	5.7
Ofloxacin	432	90.9	11	2.4	32	6.7
Amphotericin B	9	81.8	1	9.1	1	9.1
Fluconazole	10	90.9	1	9.1	-	-
Itraconazole	11	100.0	-	-	-	-

Sensitive – growth delay of more than 18 mm; low-sensitive-growth delay of 11-18 mm; insensitive-growth delay of less than 10 mm – negative result

As shown in Table-7, the highest antimicrobial activity was observed with enrofloxacin, cephalexin, norfloxacin, Cobactan, and ofloxacin, with the number of microbial isolates sensitive to these antimicrobial drugs being 466 (98.1%), 455 (95.8%), 440 (92.6%), 438 (92.2%), and 432 (90.9%), respectively, among the total number of examined strains.

The most resistant isolates of microorganisms were not sensitive to methicillin, benzylpenicillin, streptomycin, and tetracycline, with the number of isolates being 215 (45.3%), 136 (28.6%), 123 (25.8%), and 102 (21.5%), respectively, among the total number of isolated bacteria.

The isolated fungi exhibited different sensitivities to antimycotics. The most effective antimycotic agent was itraconazole that demonstrated activity against all the 11 (100.0%) isolated fungi. Moreover, 90.9% of fungal isolates were sensitive to fluconazole, and 81.8% of the representatives of *Candida* fungi were sensitive to amphotericin B.

## Conclusion

The microbial profile of the udder secretions of cows with mastitis is extremely diverse and heterogeneous. From 103 cows with udder inflammation, 486 pathogenic and conditionally pathogenic microorganisms were isolated, which belonged to 11 genera. Mastitis in cows is not caused by a single pathogen but by microbial associations, which included two to seven isolates. Only large-scale microbiological investigations isolating all the joints of parasitocenosis can provide the correct etiological picture of this disease, thus leading to its effective control approaches. Superficial bacteriological studies that do not consider the entire microbial profile of the disease will not lead to success in its treatment [35,38-41].

A relatively high percentage of isolates of microorganisms in cows with mastitis have the ability to form biofilms, indirectly characterizing their pathogenic properties. In contrast, the low ability to form biofilms in the isolated representatives of lactobacilli indicated the failure of colonization resistance of the body. It is possible that a gap in this natural barrier, which has evolved, led to the emergence of a pathological process.

The basis of a rational antibiotic therapy is the selection of antimicrobial drugs that are effective against the isolated pathogen, and it is impossible to determine this without laboratory data on its sensitivity to antibiotics. Moreover, it is important to determine the sensitivity of pathogens to antimicrobial agents to monitor the changes in their sensitivity and the formation of new determinants of resistance and for the identification of new antibiotic types in this biogeocenosis. The results of this study showed that the isolated microorganisms have different and far from heterogeneous sensitivity to the action of antimicrobial drugs. This makes it difficult to use these tools for achieving effective control of mastitis in cows, which is frequently caused by pathogenic associations. Therefore, there exists a need to explore novel and more effective methods to combat this disease.

## Authors’ Contributions

PR, NS, and YV had the original idea for the study and designed the study. SS collected the samples. SE and DB were responsible for data analysis and data cleaning. SK, OK, AK, and EV drafted the manuscript. The final draft manuscript was revised by all authors. All authors read and approved the final manuscript.
